# Levels of Physical Activity Are Associated With the Motivational Climate and Resilience in University Students of Physical Education From Andalucía: An Explanatory Model

**DOI:** 10.3389/fpsyg.2019.01821

**Published:** 2019-08-06

**Authors:** Ramón Chacón-Cuberos, Manuel Castro-Sánchez, José Antonio Pérez-Turpin, Eva María Olmedo-Moreno, Félix Zurita Ortega

**Affiliations:** ^1^Department of Research Methods and Diagnosis in Education, University of Granada, Granada, Spain; ^2^Department of Didactics of Musical, Plastic and Corporal Expression, University of Granada, Granada, Spain; ^3^Department of Didactic General and Specific Training, University of Alicante, Alicante, Spain

**Keywords:** resilience, motivational climate, physical activity, sport, university

## Abstract

**Background:**

The practice of Physical Activity (PA) is a key factor for the improvement of physical and mental health, making the study of the motivational processes that take part in the development of active lifestyles of interest.

**Methods:**

This cross-sectional study was conducted on 775 university students of Physical Education (PE) from Spain. This research aims to develop an explanatory model for the relationships between motivational climate and resilience according to the level of PA, using structural equations analysis. The main instrument used were the Perceived Motivational Climate in Sport (PMCSQ-2) and the Connor-Davidson Resilience Scale (CD-RISC).

**Results:**

A negative relationship was observed between task-oriented climate (TC) and ego-oriented climate, which acquired greater correlation strength in the respondents who did less PA. Likewise, a positive relationship was obtained between TC and resilience, which was higher in participants who did more than 3 h of weekly PA. Finally, it was observed that resilience was highly correlated with personal competence, tenacity and control capacity in the most active respondents.

**Conclusion:**

The importance of promoting task-oriented motivational climates in PA is highlighted, since this could develop a better resilience capacity in university students and will favor the tolerance to adversity and the positive acceptance of changes.

## Introduction

Given the benefits produced for physical and mental health, there’s been an expansion in recent decades in the promotion of healthy lifestyles (Ekelund et al., 2016; Lewis et al., 2017). One of the main ways to achieve this aim is the practice of Physical Activity (PA). This is defined as all body movements that involve an energy expenditure, while the practice of physical exercise implies a prescription of PA with a specific load and rest periods. Moreover, sport is a more specific concept that can be defined as a regulated and institutionalized PA that can involve competition in different ways. Thus, an adequate prescription of these habits will provide multiple benefits at the physiological level, and its promotion is essential from an early age (Bouchard et al., 2018). Specifically, World Health Organization [WHO] (2010) recommends performing at least 150 min of moderate PA or 75 min of intense PA per week in adults aged between 18 and 64 years old. This recommendation is especially important in university students, which are approaching adulthood (Arnett and Tanner, 2016). This stage is a period of labor and social complexity, in which several harmful habits can arise due to peer influence. Some of these habits include the consumption of legal drugs, sedentary digital leisure or unhealthy food intakes (Chacón-Cuberos et al., 2018a; Erturan-Ilker et al., 2018).

Among the benefits provided by positive habits figures a decrease in the risk of suffering from diseases such as osteoporosis, cancer or obesity (Warburton and Bredin, 2017). PA produces an improvement in insulin sensitivity, develops cardiorespiratory fitness through adaptations in the cardiovascular system and helps decrease the percentage of fat mass (Fransson et al., 2018; López-Sánchez et al., 2018). In addition, PA also generates a multitude of benefits at a cognitive level, which are the focus of this research work. For example, Sink et al. (2015) demonstrated that older adults who followed an active lifestyle based on PA for 24 months improved cognitive function and reaction time. Likewise, Plotnikoff et al. (2015), Donnelly et al. (2016) revealed that adolescents who performed physical exercise improved their cerebral neuroplasticity, attention capacity and academic performance. In fact, the practice of PA is associated with improvements in several psychosocial factors, such as self-esteem, perceived well-being, self-concept or resilience (Kavosi et al., 2015; Plotnikoff et al., 2015; Prakash et al., 2015).

Due to the positive effects for the health linked to the practice of PA, it is interesting to study the motivational processes that promote its practice. The Self-Determination Theory (Ryan and Deci, 2017; Chacón-Cuberos et al., 2018b) and the Achievement Goals Theory (Mascret et al., 2015; Chacón-Cuberos et al., 2018a) represent the two main models that explain the development of behaviors in the sport context. The first theory states that the level of motivation linked to a behavior establishes a continuum which varies from higher to lower level of self-determination. The most self-determined area includes more autonomous and controlled forms of motivation such as intrinsic motivation, while amotivation is found in the less self-determined area. In the middle zone the extrinsic motivation can be found, which is characterized by behaviors which are done in order to obtain some separable outcome or rewards. It is also important to point out that this level of self-determination will be mediated by three basic components, such as competence, autonomy, and relationship with others (Ryan and Deci, 2000; Gorelik et al., 2018). Briefly, the need of competence is linked to the ability to control actions effectively in order to obtain the desired result. The need for autonomy is associated with the desire of the person to build and determine their own behavior and modify it according to the need of the context. Finally, the need of relationship is linked to behaviors that improve the social world of the athlete, integrating a socializing component (Ryan and Deci, 2017; Erturan-Ilker et al., 2018).

The second model establishes that the goals fixed by a person in the sport practice depend on the perception that people have of their abilities and skills (Harwood et al., 2015; Gorelik et al., 2018; Castro-Sánchez et al., 2019). The motivational climate is defined as the set of social and contextual signals through which social agents are related, which define the keys to success or failure (Moreno et al., 2008). Thus, two types of motivational climates can be developed as a result of the relationships between peers, rivals, coaches and other agents; the task-oriented motivational climate and the ego-oriented motivational climate. Moreover, several authors have demonstrated the link between the motivational climate and the theory of self-determination (Moreno et al., 2008; Jaakkola et al., 2017). The task-oriented climate (TC) is associated with intrinsic motivations that are related to teamwork, learning new skills and enjoying the PA. On the contrary, the ego-oriented climate (EC) is related to extrinsic motivations linked to competition and obtaining better results than rivals in sports practice (Chacón-Cuberos et al., 2018b; Monteiro et al., 2018; Cordo-Cabal et al., 2019).

Under this perspective, several studies have shown how the development of TCs in sport favors the promotion of healthy habits, while the ego-oriented motivational climate is associated with greater non-adaptive behaviors (Harwood et al., 2015; Chacón-Cuberos et al., 2018b). As an example of this basis, Reinboth and Duda (2006) show how people who practice collective sports, whose coach generates a task-oriented motivational climate, have higher levels of well-being. Similarly, Chacón-Cuberos et al. (2018a) reveal that the mastery climate is related to higher levels of PA and a better quality diet, while the EC is associated with greater alcohol consumption. Finally, and taking into account other psychological factors, Vitali et al. (2015) show that TC acts as a protective factor against burnout situations, as well as helping the development of resilience capacity.

The resilience capacity has been widely studied in the sport and academic context (Connor and Davidson, 2003; Doll et al., 2014; Vitali et al., 2015). This psychosocial factor refers to the ability to overcome situations of adversity (Connor and Davidson, 2003). In fact, resilience is closely associated with situations of stress and anxiety, since these negative factors are generated in situations in which an individual is not perceived with sufficient competence to overcome an event (Olmedilla et al., 2018; Zurita-Ortega et al., 2018). Therefore, it is of special interest to carry out actions that improve resilience capacity, since it acts as a protective factor against many vital experiences (Al-Haramlah, 2018). Sarkar and Fletcher (2014) show how the development of personal factors such as confidence, self-determined motivation and social support helps improve resilience and reduce the effect of stressors in the sport context. Moreover, Galli and Gonzalez (2015) highlight the importance of developing intervention programs based on the development of personal beliefs and the ability to control negative situations. In addition, and considering the association with the theoretical framework developed, it is interesting to know its association with PA and motivation. For example, the study conducted by Hegberg and Tone (2015) reveals through a linear regression analysis how the PA and the perceived resilience are positively related, which is mediated by the trait anxiety levels. Furthermore, Ka et al. (2015) point out in a study conducted in young people from China how PA was related to mental wellbeing, self-efficacy and resilience, which shows the potential of this psychosocial factor and its association with healthy habits.

This research considers the motivational orientations involved in PA and the relationships of the motivational climate on the resilience capacity in different context. The present study sought to answer the following research question: are there differences in the relationship between motivational climate and the capacity of resilience according to the level of PA done? Given the findings of previous research the following hypotheses are proposed:

• Hypothesis 1 (H1): TC will be directly related to the resilience capacity, while EC will be inversely associated with resilience.
• Hypothesis 2 (H2): University students who follow a more active lifestyle will show a stronger relationship between the TC and resilience capacity.

Thus, the following aims are set in the present study: (a) to develop an explanatory model about the relationships between motivational climate, resilience and its different indicators; (b) to contrast the structural equation model (SEM) developed according to the level of PA through multi-group analysis.

## Materials and Methods

### Subjects and Design

This study presents a cross-sectional design with a single measurement in a single group. The study sample consisted of 775 university students of Physical Education (PE) from the eight provinces of the Autonomous Community of Andalusia (Spain), with 58.7% (*n* = 455) men and a 41.3% (*n* = 320) women. The age of the respondents was between 21 and 35 years old (22.22 ± 3.76). A total of 1167 students were enrolled in the mention of PE (degree in Primary Education) during the academic year 2016/2017 (data provided by the different universities). Considering the university centers that accepted to participate in this study and the selection criteria [(1) To study PE degree; (2) To attend regularly to university -at least 75% of attendance in class considering the check-list of their professors-; (3) Not to suffer from important pathologies], a sample of 829 subjects was considered using simple random sampling. A total of 54 questionnaires had to be eliminated because they were wrongly completed, obtaining a final sample of 775 university students. It can be considered that a representative sample was obtained for the studied population (university students of PE), with a sampling error of 0.05 and a CI of 95.5%. All the participants gave written informed consent.

### Measures

This study used some main instruments as described below.

Perceived Motivational Climate in Sport Questionnaire (PMCSQ-2) (Walling et al., 1993). The Spanish version validated by González-Cutre et al. (2008) was used. This instrument allows to evaluate the motivational climate in sport and it is composed of 33 five-point items ranging from 1 to 5 (1 = Strongly Disagree; 5 = Strongly Agree). This scale establishes two dimensions (TC and ego-oriented climate), each containing three factors. These are Effort/Improvement, Cooperative learning, and Important role for the TC and Punishment for mistakes, Member rivalry, and Unequal recognition for the ego-oriented climate. This instrument has an acceptable value for Cronbach’s alpha (α = 0.85), showing an appropriate internal consistency. For each dimension, TC showed an excellent value of α = 0.92, while EC ghas a value of α = 0.93.

Connor–Davidson Resilience Scale (CD-RISC), developed by Connor and Davidson (2003) and validated into Spanish by Olmo et al. (2017), which allows the assessment of the resilience capacity of respondents. This instrument is composed of 25 five-point items ranging from 0 to 4 (0 = I totally disagree; 4 = I totally agree). The 25 items conform to five factors related to resilient behavior, such as personal ability and tenacity, confidence and tolerance for adversity, positive acceptance of changes, capacity of control and spiritual influence. This instrument has an excellent reliability in the present study, showing an alpha value of α = 0.90. For each dimension the values were: Personal competence (α = 0.85), Tolerance to adversity (α = 0.71), Positive acceptance to changes (α = 0.72), Control capacity (α = 0.65), and Spirituality (α = 0.59).

International Physical Activity Questionnaire (IPAQ) (Craig et al., 2003), validated into Spanish by Mantilla and Gómez-Conesa (2007), allowed the evaluation of levels of PA done in the last week by respondents. This scale is scored through a five-point Likert scale with seven items (0 = Never; 4 = Always) obtaining a summation that establishes the global level of PA in the last 7 days. Subsequently, a variable of categorical type was created, which determined whether the participants performed more than 3 h per week of PA outside academic hours. This variable of dichotomous type (1 = Yes; 2 = No) was used for the multi-group analysis of the two structural equation models. This research obtained an acceptable reliability in this research (α = 0.86).

### Procedure

First, the collaboration of the respondents was requested through an informative letter created by the Corporal Area of the University of Granada. This was provided to the university students who attended the Mention of PE of the degree of Primary Education of the eight Andalusian provinces. This document detailed the proposal of the research, as well as the objectives of the same. In addition, the informed consent of the participants was requested. This was obtained and written.

The application of the described instruments was subsequently done. A total of 829 university students took part in the study. Data collection was done during school hours in the different university centers without any type of incident. Moreover, researchers were present in order to ensure the correct application of the instruments. A total of 54 questionnaires had to be eliminated because they were wrongly filled out, leaving a final sample of 775 university students.

The anonymity of all subjects has been respected, as well as the Declaration of Helsinki of 1975 for studies with humans. Similarly, the Research Ethics Committee of the University of Granada (Spain) approved this study (462/CEIH/2017). Respondents participated voluntarily in this research.

### Statistical Analysis

**FIGURE 1:**
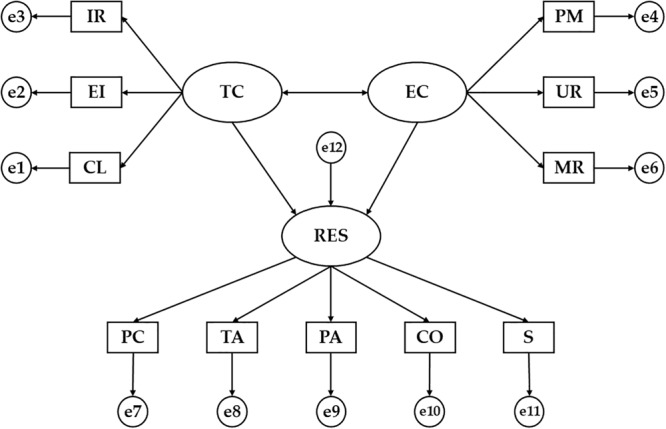
Theoretical Model. TC, task-oriented climate; IR, important role; EI, effort/improvement; CL, cooperative learning; EC, ego-oriented climate; PM, punishment for mistakes; UR, unequal recognition; MR, member rivalry; RES, resilience; PC, personal competence; TA, tolerance to adversity; PA, positive acceptance to changes; CO, control capacity; S, spirituality.

First, the IBM SPSS^®^23.0 (IBM Corp., Armonk, NY, United States) software was used to check the normal distribution of the sample through the values of asymmetry and kurtosis of the items of the scales. In this case, no value equal to or greater than 2 was obtained, showing a normal distribution. In addition, frequencies and means were used for the basic descriptive and the *T*-test to verify the existence of statistically significant differences between variables. On the other hand, the IBM AMOS^®^23 (IBM Corp., Armonk, NY, United States) software was used to analyze the relationships between the involved constructs of the structural model. Once the theoretical model is developed, a SEM is carried out considering the relationships of the matrix from a multi-group analysis according to the level of practice of PA. The SEM is made up of 11 observable variables and three latent variables to determine the indicators (Figure 1). In these, explanations of the associations between the latent variables are formulated from the observed relationships. Likewise, measurement errors (circles) are included in the observable variables so that they are directly controlled. The arrows are lines of relationships between the variables and these are interpreted as regression coefficients.

The SEM showed in Figure 1 is composed of three latent variables (ovals) and 11 observed variables (squares). Task Climate (TC) and Ego Climate (EC) are the latent and exogenous variables. These two exogenous variables were inferred by the following six observed variables: Important Role (RI), Cooperative Learning (CL), and Effort/Improvement (EI) for Task Climate (TC), and Member Rivalry (MR), Unequal Recognition (UR), and Punishment for Mistakes (PM) for Ego Climate (EC). Another latent variable was Resilience (RES), which was inferred by Personal Competence (PC), Tolerance to Adversity (TA), Positive Acceptance to changes (PA), Control Capacity (CO), and Spirituality (S).

The method of maximum likelihood (ML) was used to estimate relationships between variables. We chose this method because it is consistent, unbiased and invariant to types of scale, given variables with a normal distribution. Model fit was examined to verify the compatibility of the proposed model and the empirical information gathered. Goodness of fit was tested using a number of indices described (Barrett, 2007). Chi-squared analysis followed when non-significant p-Values indicated a good model fit. Comparative fit index (CFI), normalized fit index (NFI) and increase fit index (IFI) values higher than 0.90 indicate acceptable model fit while values higher than 0.95 indicate excellent model fit. Root mean square error of approximation (RMSEA) values below 0.08 indicate acceptable model fit while values below 0.05 indicate excellent model fit.

## Results

First, descriptive data of the sample are shown in relation to the variables (motivational climate and resilience), considering the gender [men = 58.7% (*n* = 455); women = 41.3% (*n* = 320)] and the practice of more than 3 h per week of PA [+3 h/week = 74.1% (*n* = 574); -3 h/week = 25.9% (*n* = 201)]. Specifically, Table 1 shows the differences between men and women for all sub-dimensions of the ego-oriented climate, being higher in men. Considering the practice of weekly PA, it was observed that those who practiced more than three weekly non-teaching hours had higher scores in the global TC, cooperative learning, unequal recognition and rivalry between members.

**Table 1:** Levels of motivational climate according to gender and PA.

Motivational climate	Gender	Mean ± SD	Motivational climate	PA	Mean ± SD
TC	Men	3.90 ± 0.66	TC^∗^	+3 h/week	3.91 ± 0.67
	Women	3.87 ± 0.65		–3 h/week	3.80 ± 0.59
CL	Men	3.90 ± 0.79	CL^∗^	+3 h/week	3.92 ± 0.81
	Women	3.89 ± 0.81		–3 h/week	3.78 ± 0.80
EI	Men	3.93 ± 0.67	EI	+3 h/week	3.89 ± 0.65
	Women	3.86 ± 0.62		–3 h/week	3.83 ± 0.58
IR	Men	3.85 ± 0.76	IR	+3 h/week	3.88 ± 0.81
	Women	3.87 ± 0.80		–3 h/week	3.82 ± 0.69
EC^∗^	Men	2.43 ± 0.78	EC	+3 h/week	2.30 ± 0.82
	Women	2.22 ± 0.84		–3 h/week	2.25 ± 0.83
PM^∗^	Men	2.18 ± 0.80	PM	+3 h/week	2.07 ± 0.80
	Women	2.03 ± 0.83		–3 h/week	2.12 ± 0.91
UR^∗^	Men	2.53 ± 0.98	UR^∗^	+3 h/week	2.39 ± 1.02
	Women	2.29 ± 1.04		–3 h/week	2.27 ± 1.03
MR^∗^	Men	2.71 ± 0.78	MR^∗^	+3 h/week	2.54 ± 0.86
	Women	2.44 ± 0.86		–3 h/week	2.45 ± 0.78

*TC, task-oriented climate; IR, important role; EI, effort/improvement; CL, cooperative learning; EC, ego-oriented climate; PM, punishment for mistakes; UR, unequal recognition; MR, member rivalry; SD, standard deviation. ^∗^Statistically significant differences (*p* < 0.05).*

Table 2 shows the levels of resilience based on gender and the practice of PA. Statistically significant differences were observed in global resilience, personal competence and positive acceptance to change, with higher scores in men. In addition, statistically significant differences were revealed with regards to the levels of PA, with higher scores for those respondents who do more PA in global resilience, personal competence and tolerance to adversity.

**Table 2:** Levels of resilience according to gender and PA.

Resilience	Gender	Mean ± SD	Resilience	PA	Mean ± SD
RES^∗^	Men	3.05 ± 0.56	RES^∗^	+3 h/week	3.01 ± 0.50
	Women	2.96 ± 0.46		–3 h/week	2.90 ± 0.48
PC^∗^	Men	3.21 ± 0.65	PC^∗^	+3 h/week	3.16 ± 0.60
	Women	3.10 ± 0.57		–3 h/week	3.02 ± 0.56
TA	Men	2.98 ± 0.60	TA^∗^	+3 h/week	2.88 ± 0.55
	Women	2.80 ± 0.53		–3 h/week	2.79 ± 0.59
PA^∗^	Men	3.18 ± 0.62	PA	+3 h/week	3.18 ± 0.59
	Women	3.14 ± 0.59		–3 h/week	3.00 ± 0.64
CO	Men	3.00 ± 0.71	CO	+3 h/week	3.03 ± 0.69
	Women	3.02 ± 0.67		–3 h/week	2.94 ± 0.64
S	Men	2.42 ± 0.96	S	+3 h/week	2.39 ± 0.89
	Women	2.40 ± 0.83		–3 h/week	2.46 ± 0.77

*RES, resilience; PC, personal competence; TA, tolerance to adversity; PA, positive acceptance to changes; CO, control capacity; S, spirituality; SD, standard deviation. ^∗^Statistically significant differences (*p* < 0.05).*

Subsequently, the SEM was carried out, including the motivational climate and resilience, through a multi-group analysis according to the practice of PA. The path model showed correct fit indices in the parameters analyzed. *P*-value reveals a statistically significant value (χ^2^ = 210.200, *df* = 82, *p* < 0.001). This index should not be interpreted in a standardized way due to its sensitivity to sample size. In this way, other fit indices were included as established by Marsh (2007). The NFI revealed an acceptable value of 0.94, while the CFI and the Increment Fit Index (IFI) showed excellent an value of 0.96 for both parameters. Moreover, an acceptable value of 0.05 was obtained for the RMSEA.

**FIGURE 2:**
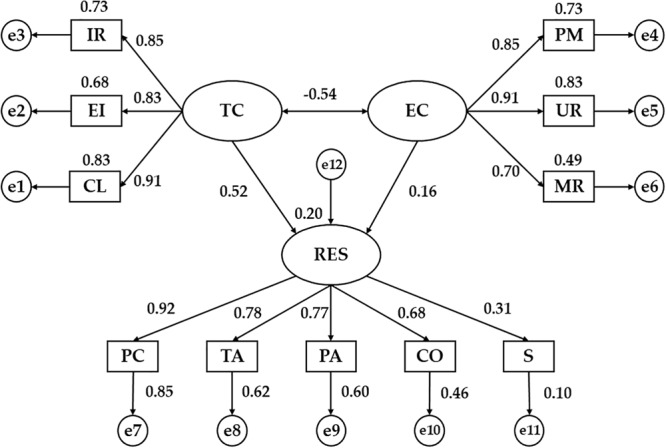
Structural Equation Model for active students (>3 h/week of PA). TC, task-oriented climate; IR, important role; EI, effort/improvement; CL, cooperative learning; EC, ego-oriented climate; PM, punishment for mistakes; UR, unequal recognition; MR, member rivalry; RES, resilience; PC, personal competence; TA, tolerance to adversity; PA, positive acceptance to changes; CO, control capacity; S, spirituality.

First, it is showed the SEM for respondents considered physically active (more than 3 hours of PA/week) (Figure 2 and Table 3). Statistically significant differences were obtained at the *p* < 0.001 level in the associations given between the two dimensions of motivational climate and its indicators. In this sense, the indicator that had the greatest influence on TC was CL (*b* = 0.91), while the indicator that obtained the lowest regression weight was the Effort/Improvement (EI) (*b* = 0.83). For EC the highest correlation was showed by the UR (*b* = 0.91), while the indicator with the least influence was the Rivalry between members (MR) (*b* = 0.70). Likewise, the TC and EC were inversely related (*b* = -0.54, *p* < 0.001).

**Table 3:** Regression weights and standardized regression weights in active students (>3 h/week of PA).

			RW				SRW
	Relationship between variables		EST	SE	CR	*P*-values	EST
RES	←	TC	0.388	0.052	7.398	^∗∗∗^	0.517
RES	←	EC	0.131	0.056	2.349	^∗^	0.161
CL	←	TC	1.000	–	–	^∗∗∗^	0.912
EI	←	TC	0.724	0.036	19.942	^∗∗∗^	0.826
IR	←	TC	0.940	0.045	21.058	^∗∗∗^	0.855
PM	←	EC	1.000	–	–	^∗∗∗^	0.853
UR	←	EC	1.368	0.074	18.375	^∗∗∗^	0.910
MR	←	EC	0.891	0.063	14.241	^∗∗∗^	0.698
PC	←	RES	1.000	–	–	^∗∗∗^	0.920
TA	←	RES	0.789	0.044	17.831	^∗∗∗^	0.784
PA	←	RES	0.827	0.047	17.441	^∗∗∗^	0.773
CO	←	RES	0.857	0.059	14.462	^∗∗∗^	0.681
S	←	RES	0.505	0.088	5.708	^∗∗∗^	0.313
EC	↔	TC	–0.273	0.035	–7.712	^∗∗∗^	–0.542

*TC, task-oriented climate; IR, important role; EI, effort/improvement; CL, cooperative learning; EC, ego-oriented climate; PM, punishment for mistakes; UR, unequal recognition; MR, member rivalry; RES, resilience; PC, personal competence; TA, tolerance to adversity; PA, positive acceptance to changes; CO, control capacity; S, spirituality; SRW, standardized regression weights; SE, estimation of Error; CR, critical ratio; RW, regression weights; EST, estimation. ^∗^*p* < 0.05 and ^∗∗∗^*p* < 0.001.*

Statistically significant differences (*p* < 0.001) were observed between resilience capacity (RES) and all its indicators, showing direct relationships. The variable with the highest influence was Personal competence and tenacity (PC) (*b* = 0.92), while the one with the lowest regression weight was Spirituality (S) (*b* = 0.31). Moreover, the relationships between the motivational climate and the RES were positive and direct, acquiring a greater correlation strength for the TC (*b* = 0.52, *p* < 0.001) than for the EC (*b* = 0.16, *p* < 0.05).

**FIGURE 3:**
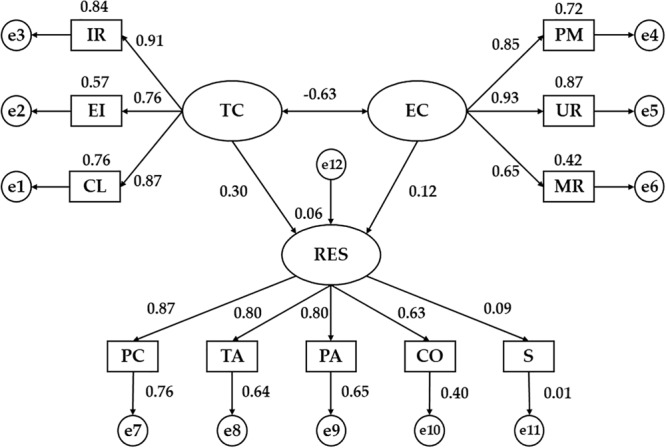
Structural Equation Model for non-active students (<3 h/week of PA). TC, task-oriented climate; IR, important role; EI, effort/improvement; CL, cooperative learning; EC, ego-oriented climate; PM, punishment for mistakes; UR, unequal recognition; MR, member rivalry; RES, resilience; PC, personal competence; TA, tolerance to adversity; PA, positive acceptance to changes; CO, control capacity; S, spirituality.

Figure 3 and Table 4 show the regression weights of the SEM designed for those university students who follow a less active lifestyle (less than 3 h of PA/week). Statistically significant differences were obtained for the relationships given between all the indicators and the two dimensions of the motivational climate (*p* < 0.001) as showed by the previous SEM. In this case, the indicator with the highest regression weight for the TC was the RI (*b* = 0.91), while the variable with the least association was the EI (*b* = 0.76). The highest correlation was shown with UR (*b* = 0.93) for the CE dimension, with the least regression weight for MR (*b* = 0.65).

**Table 4:** Regression weights and standardized regression weights in non-active students (<3 h/week of PA).

			RW				SRW
	Relationship between variables		EST	SE	CR	*P*-values	EST
RES	←	TC	0.215	0.072	2.970	^∗∗^	0.300
RES	←	EC	0.086	0.071	1.220	0.222	0.122
CL	←	TC	1.000	–	–	^∗∗∗^	0.871
EI	←	TC	0.633	0.047	13.525	^∗∗∗^	0.758
IR	←	TC	1.047	0.061	17.176	^∗∗∗^	0.915
PM	←	EC	1.000	–	–	^∗∗∗^	0.851
UR	←	EC	1.383	0.089	15.550	^∗∗∗^	0.930
MR	←	EC	0.729	0.068	10.676	^∗∗∗^	0.650
PC	←	RES	1.000	–	–	^∗∗∗^	0.871
TA	←	RES	0.947	0.069	13.698	^∗∗∗^	0.802
PA	←	RES	0.916	0.067	13.719	^∗∗∗^	0.804
CO	←	RES	0.839	0.083	10.051	^∗∗∗^	0.631
S	←	RES	0.146	0.110	1.329	0.184	0.094
EC	↔	TC	–0.345	0.050	–6.859	^∗∗∗^	–0.633

*TC, task-oriented climate; IR, important role; EI, effort/improvement; CL, cooperative learning; EC, ego-oriented climate; PM, punishment for mistakes; UR, unequal recognition; MR, member rivalry; RES, resilience; PC, personal competence; TA, tolerance to adversity; PA, positive acceptance to changes; CO, control capacity; S, spirituality; SRW, standardized regression weights; SE, estimation of error; CR, critical ratio; RW, regression weights; EST, estimation. ^∗∗^*p* < 0.01 and ^∗∗∗^*p* < 0.001.*

In a similar line, statistically significant relationships (*p* < 0.001) are shown for all the indicators of the RES except for the S. The variable with the greatest regression weight was the PC (*b* = 0.87), while the one with the least influence was the CO (*b* = 0.63). Likewise, statistical associations were only observed for the relationship between TC and RES in the university students linked to a lower level of PA, showing a positive and direct association (*b* = 0.30, *p* < 0.01).

## Discussion

This research aims to analyze the relationships between motivational climate in sport and resilience capacity in university students using a SEM with multi-group analysis. This association is analyzed according to the level of PA in order to verify the relationship between these variables depending on the practice of more than 3 h per week of PA. Supporting this study, other similar researches are those carried out by Lee and Loke (2005), Schnettler et al. (2015), Chacón-Cuberos et al. (2018a), Zurita-Ortega et al. (2018), which address physical and mental health in university students as a risk group for harmful behaviors.

First, the influence exerted by the indicators of each dimension of the motivational climate is analyzed. The SEM showed that CL was the most influential indicator in the TC for students who practice more PA. The IR was the indicator with the higher regression weight for the respondents who did less than 3 h per week of PA. These results can be explained by the findings of Parish and Treasure (2003), who demonstrate how the most self-determined motivations favor higher levels of PA, which is linked to the TC as it is shown in the present research. Moreover, teamwork is more associated with intrinsic motivations given the hedonistic and social component involved in sports practice, which makes university students want to maintain this habit (Monteiro et al., 2018; Montero-Cobo et al., 2018). Nevertheless, the IR is established as an indicator that can be associated with both intrinsic and extrinsic motivations (Hodge and Gucciardi, 2015). For this reason, university students who are related to extrinsic motivations such as competition, practice sports with less frequency (Winter and Collins, 2015).

According to the ego-oriented climate, the UR obtained the greatest regression weight for active and non-active students, although this showed a higher score in the non-active students despite the difference being minimal. This lower influence can be justified by the premises previously explained, given that university students who practice less PA are linked to external motivations associated with specific rewards and competition (Winter and Collins, 2015; McLaren et al., 2017). Specifically, Castro-Sánchez et al. (2018) established that the sport practice associated with competition can be linked to negative emotions that emerge from unwanted results, such as defeat, anxiety or frustration. This causes these individual to stop practicing sport due to these negative feelings, which make them less physically active (McLaren et al., 2017; Castro-Sánchez et al., 2018). Likewise, this type of motivation can be linked to non-adaptive behaviors, such as the consumption of harmful substances, the intensive use of technological devices or poor diets (Chacón-Cuberos et al., 2018b).

Personal competence and control capacity acquired a slightly higher regression weight for resilience in active people. These findings highlight that PA can be linked to an improvement of personal competence through tenacity and the ability to overcome negative situations. It is because the practice of PA generates endorphins and reduces stress, helps maintain levels of effort, allows the setting of attainable goals and favors the development of self-determined motivations (Mandolesi et al., 2018; Zurita-Ortega et al., 2018). In addition, Shoenfelt (2016) establish how the practice of sport improves the control capacity, since the athlete will learn to fight against situations of adversity such as defeat, and negative emotions such as anxiety, frustration or fear. In contrast, tolerance to adversity and positive acceptance of changes were the indicators more related to resilience in the respondents who practice fewer than 3 h of PA per week. This situation may be due to a lower capacity of these students to maintain behaviors when establishing goals, being less persistent and forced to accept the new events (Morgan et al., 2017). Thus, the respondents who practice more PA are more optimistic and empathic, which makes them more resilient without the need to achieve their objectives (Laborde et al., 2016).

The relationship between motivational climate and resilience revealed a positive relationship between TC and resilience capacity, which was higher in the more active respondents. From these results, it can be inferred that those students who practice more PA linked to intrinsic motivations such as hedonism, learning improvement or socialization, are more resilient. Specifically, Vitali et al. (2015) demonstrate how coaches who develop task-oriented motivational climates help improve the level of achievement in athletes, sports devaluation and burnout levels, acting as a protective factor and improving resilience. On a similar note, Martin and Dowson (2009) emphasize that positive psychology, which has certain elements in common with self-determined motivations, can be positively related to resilience. Specifically, it highlights the importance of favoring adaptive behaviors within the cognitive sphere such as goals oriented toward mastery, self-efficacy and the value of work (Crane et al., 2017; Chacón-Cuberos et al., 2018b). Likewise, within the field of behavior, emphasis is placed on developing persistence, planning and the ability to organize work tasks (Martin and Dowson, 2009; Secades et al., 2016).

The EC showed a lower association with resilience, and only in active people due to the relationship that exists between both motivational climates in those people who practice sports frequently. Specifically, Matosic et al. (2017) remember how the promotion of both motivational climates generates a positive effect on the adherence of university students to sports practice, justifying these findings. Nevertheless, the development of intrinsic motivations should not be neglected, since several works establish how motivational climates oriented exclusively to the ego are linked to situations of self-handicap, disgust, poor control and avoidance of failure (Martin and Dowson, 2009; Verner-Filion et al., 2014). This is due to situations in which the desired results are not achieved, when there is excessive competition or when these two factors lead to high levels of state anxiety, stress or sports burnout (Smith et al., 2007). Therefore, it can be concluded that the promotion of motivational climates oriented to the task could favor the resilient capacity and act in a preventive way with negative emotions in sports.

It is important to establish the main limitations of this study. The first is associated with the study design, since a cross-sectional research do not allow to show cause-effect relationships. However, the SEM is useful to know the associations between the psychosocial variables which have been studied. Moreover, multi-group analysis is used in order to compare the regression weights of this relationship according to a dichotomous variable as the practice of PA. Another limitation is linked to the study sample, since only university students who attend PE degrees were considered. Consequently, it can be established that this sample already has a relatively high level of PA. Therefore, there is a need to replicate this study in other types of university students, since students from other degrees may be more sedentary and may develop more harmful habits such as a poor diet or substance abuse. In this way, we could expand the vision of the existing relationships between PA level, resilience and motivation in sports as future perspectives. Finally, it would also be interesting to include other psychological factors associated with resilience, such as self-concept or burnout syndrome.

## Conclusion

Considering the research question, it can be established that resilience is associated with the type of motivational climate in sport and the levels of PA of university students. Nevertheless, this relationship depends on the type of motivational climate, showing different types of influence in this capacity. Therefore, we should point out about the hypothesis:

• Hypothesis 1 (H1) was partially fulfilled, since TC was positively related to resilience. However, EC was not inversely associated with resilience, showing a positive relationship in respondents who practice more PA.
• Hypothesis 2 (H2) was fulfilled. The university students who practice more than 3 h per week of PA showed a stronger relationship between TC and resilience capacity.

As a main conclusion, it can be established that the practice of PA could be associated with more favorable motivational climates and levels of resilience capacity in university students. Specifically, respondents who do more than 3 h of PA per week showed slightly higher levels of TC, which were linked to a greater resilience. This factor was mainly influenced by a personal competence and the capacity of control.

## Data Availability

The datasets for this manuscript are not publicly available because the reason is that they are part of a project and belong to the research group. Requests to access the datasets should be directed to the corresponding author.

## Ethics Statement

All procedures performed in studies involving human participants were in accordance with the ethical standards of the institutional and/or national research committee and with the 1964 Helsinki Declaration and its later amendments or comparable ethical standards. Written informed consent was obtained from all individual participants included in this study.

## Author Contributions

RC-C, EO-M, and FZO conceived the hypothesis of this study. FZO, JP-T, and MC-S participated in the data collection. RC-C and MC-S analyzed the data. All authors contributed to the data interpretation of the statistical analysis, and read and approved the final manuscript. RC-C, EO-M, and MC-S wrote the manuscript with the significant input from FZO and JP-T.

## Conflict of Interest Statement

The authors declare that the research was conducted in the absence of any commercial or financial relationships that could be construed as a potential conflict of interest.
